# Use of the Theoretical Domains Framework to evaluate factors driving successful implementation of the Accelerated Chest pain Risk Evaluation (ACRE) project

**DOI:** 10.1186/s13012-016-0500-9

**Published:** 2016-10-12

**Authors:** Wade Skoien, Katie Page, William Parsonage, Sarah Ashover, Tanya Milburn, Louise Cullen

**Affiliations:** 1 Royal Brisbane and Women’s Hospital, Butterfield St & Bowen Bridge Rd, Herston, QLD 4006 Australia; 2 Institute of Health and Biomedical Innovation, Queensland University of Technology, 60 Musk Avenue, Kelvin Grove, QLD Australia

**Keywords:** Implementation science, Research translation, Theoretical domains framework, Behaviour change, Low and intermediate risk chest pain

## Abstract

**Background:**

The translation of healthcare research into practice is typically challenging and limited in effectiveness. The Theoretical Domains Framework (TDF) identifies 12 domains of behaviour determinants which can be used to understand the principles of behavioural change, a key factor influencing implementation. The Accelerated Chest pain Risk Evaluation (ACRE) project has successfully translated research into practice, by implementing an intervention to improve the assessment of low to intermediate risk patients presenting to emergency departments (EDs) with chest pain. The aims of this paper are to describe use of the TDF to determine which factors successfully influenced implementation and to describe use of the TDF as a tool to evaluate implementation efforts and which domains are most relevant to successful implementation.

**Methods:**

A 30-item questionnaire targeting clinicians was developed using the TDF as a guide. Questions encompassed ten of the domains of the TDF: *Knowledge*; *Skills*; *Social/professional role and identity*; *Beliefs about capabilities*; *Optimism*; *Beliefs about consequences*; *Intentions*; *Memory, attention and decision processes*; *Environmental context and resources*; and *Social influences*.

**Results:**

Sixty-three of 176 stakeholders (36 %) responded to the questionnaire. Responses for all scales showed that respondents were highly favourable to all aspects of the implementation. Scales with the highest mean responses were Intentions, Knowledge, and Optimism, suggesting that initial education and awareness strategies around the ACRE project were effective. Scales with the lowest mean responses were Environmental context and resources, and Social influences, perhaps highlighting that implementation planning could have benefitted from further consideration of the factors underlying these scales.

**Conclusions:**

The ACRE project was successful, and therefore, a perfect case study for understanding factors which drive implementation success. The overwhelmingly positive response suggests that it was a successful programme and likely that each of these domains was important for the implementation. However, a lack of variance in the responses hampered us from concluding which factors were most influential in driving the success of the implementation. The TDF offers a useful framework to conceptualise and evaluate factors impacting on implementation success. However, its broad scope makes it necessary to tailor the framework to allow evaluation of specific projects.

**Electronic supplementary material:**

The online version of this article (doi:10.1186/s13012-016-0500-9) contains supplementary material, which is available to authorized users.

## Background

The translation of healthcare research into practice is typically slow, challenging, and often fails. Only 14 % of published research is translated into bedside practice to the benefit of patients and takes an average time of 17 years to occur [1, 2]. There is increasing pressure to translate findings from research into positive health outcomes in a shorter timeframe [3–5], driven partly by the decrease in research funding availability [6]. The quality of the evidence behind a new intervention is only one factor that influences successful translation into practice. Factors across multiple levels of healthcare from the individual patient and clinician to the organisational and policy levels also require consideration [7]. The research-practice gap has led to increased focus on implementation science, which aims to improve the uptake of research findings and evidence-based practice (EBP) into routine practice, to improve the quality and effectiveness of health care and services [8].

Implementation science is a fast growing discipline. The methods are still young and the theories heterogeneous and the practical application relatively sparse. Embedding evidence into practice is not a linear or technical process that can be achieved by individual clinical champions alone. Evidence shows implementation requires whole system change involving both the individual and organisation [9]. Theoretical frameworks can help explain why implementation efforts succeed or fail [5], but the biggest challenge influencing the implementation of new interventions is behaviour change, with many frameworks therefore focussing on human behaviour theories [10–12]. Traditionally, implementation efforts have been based on interventions that are intuitive or educational in nature, such as printed education materials, audit and feedback and reminders. However, these only have a modest effect on changing clinical practice and could be improved on by considering theories of human behaviour [12]. The Theoretical Domains Framework (TDF) developed by Michie et al. [12] used expert consensus to identify 12 domains of behaviour determinants that could be used in implementation research. Cane et al. [10], who validated and refined the TDF, also agree that understanding the principles of behavioural change and changing the behaviours of healthcare professionals is the key factor in implementation efforts, but are rarely considered when designing and evaluating implementation interventions.

The Accelerated Chest pain Risk Evaluation (ACRE) project has implemented a new intervention and translated research into practice in a much shorter timeframe than traditionally reported. The ACRE project is a state-wide clinical redesign project in Queensland, Australia, implementing an accelerated diagnostic protocol (ADP) to improve the assessment of low to intermediate risk patients presenting with chest pain to emergency departments (EDs). The project translates high-quality evidence from research conducted by our group and published in 2012 [13], into effective clinical practice. The 2-Hour Accelerated Diagnostic Protocol to Assess Patients with Chest Pain Symptoms Using Contemporary Troponins as the Only Biomarker (ADAPT) trial, was a large retrospective observational study which tested whether an ADP for patients presenting with possible cardiac chest pain, could safely identify low-risk patients that were suitable for early discharge. The ADAPT-ADP uses the Thrombolysis in Myocardial Infarction (TIMI) score, electrocardiography and 0 and 2-h values of laboratory troponin I to identify these low-risk patients. The ADAPT-ADP safely identified 20 % of patients as low risk and suitable for early discharge from the ED. Given that chest pain accounts for a significant proportion of all ED presentations and has a high hospital admission rate, accelerating the care for 20 % of this cohort may reduce burden on the healthcare system [13]. A successful, single site pilot study took place in 2013 [14], in which similar results to the ADAPT trial were achieved in practice. In an approximately 2.5-year period following this, from 2013 to 2016, 21 suitable target hospitals (determined by the availability of an on-site pathology laboratory for laboratory-based troponin testing) were engaged on a site-by-site basis. Key stakeholders at each site were initially invited to attend introductory meetings and discussions about the ACRE project and possible implementation of the ADAPT-ADP in their hospital. The ADAPT-ADP is utilised primarily in the ED, and therefore, engagement of senior ED clinicians to work with the project team in implementing the ADAPT-ADP at each site was essential. However, support from other clinicians that manage patients presenting with chest pain, such as from cardiology, general medicine and clinical measurements departments were essential to implementation, as each site had different processes, capabilities and resources for managing and following up patients with possible cardiac chest pain, which influenced how changes to practice would be made. The development and modification of clinical decision pathways to incorporate the ADAPT-ADP into local practice was a key step in implementation that required input and agreement across these multiple disciplines. Funding was obtained to allow for the employment of several part-time clinical leads and project officers to drive this implementation of the ADAPT-ADP state-wide.

Nineteen of the 21 suitable target hospitals have implemented the ADAPT-ADP and adopted new practice in the management of low-intermediate-risk chest pain patients. Time taken to implement from introductory meetings to the use of the pathway in practice varied from approximately 3 months to greater than 12 months. Greater than 20 % of ED patients presenting with chest pain have been identified as low risk and managed according to the accelerated arm of the ADAPT-ADP state-wide. This closely matches the proportion demonstrated in the original research and pilot site. Comparison of data pre- and post-implementation has demonstrated that use of the ADAPT-ADP in widespread practice has significantly decreased total hospital length of stay (by 33.4 %, from 1210 to 806 mins), decreased ED length of stay (from 230 to 213 mins), and decreased admission rates to inpatient units (by 13.1 %, from 70.4 to 57.3 %) for all patients with possible cardiac chest pain [15], which represents approximately 6 % of all ED presentations [16].

The ACRE project has successfully demonstrated implementation outcomes and translation of research into practice. However, whilst a coordinated plan was developed to implement the intervention, the design could be described as one based on ‘intuition’ rather than theory, which can limit the understanding of behaviour change processes that underlie effective interventions [10]. Therefore, this paper uses a validated theoretical framework to determine which factors influencing behaviours successfully influenced implementation to help inform future translational projects, an area that is typically challenging and with a high failure rate. The second aim of this paper is to further describe the use of the TDF as a tool to evaluate implementation efforts and add to the limited available evidence and testing on which domains and scales are most relevant to successful implementation.

## Methods

This study was an effectiveness-implementation hybrid study, performed during a large-scale redesign project of assessment processes for emergency patients with possible acute coronary syndromes. The state-wide redesign project was conducted across 19 hospitals from October 2013 to June 2016, with the implementation research conducted from October 2015 to December 2015.

### Questionnaire development

A 30-item questionnaire targeting clinicians was developed using the TDF as a guide (Table 1). The questions encompass ten of the domains of the TDF. As a starting point, generic questions were taken from Huijg et al*.* [17] who used the consensus opinion of 19 academic judges to allocate questions to TDF domains and tested the discriminant content validity of each item to ensure it measured the intended domain. The final, generic questionnaire they developed included questions under 11 of the 14 domains described by Cane at al. [10], and three domains were excluded as their study did not demonstrate discriminant content validity of the items measuring those domains (Huijg ref). They also state that the 12-domain, original version of the TDF might be more applicable in developing a TDF-based questionnaire (Huijg ref). We then drew on the interview questions and domains used by Curran et al. [18], to add to and refine the questions, as there were similarities in the intervention they described to the ACRE implementation project. These include the assessment of implementation outcomes using the TDF *post-implementation*; the intervention was implementing a ‘*clinical decision rule*’; and the change efforts were also in emergency departments. One domain was subsequently excluded from our questionnaire—*Emotion*, as we did not think the example questions from Huijg et al*.* [17] relevant in our questionnaire. Curran et al. [18] used this domain; however, they determined that this was not one of the domains likely to explain physician response to implementation of their clinical decision rule. Finally, questions were adapted to address factors that we believed were relevant to the implementation of our project, based on the ACRE team beliefs or from informal stakeholder feedback. Three questions under each of the domains were chosen to be included in the final survey, except the domains *Environmental context and resources* and *Intentions* which had four and two questions, respectively, as this reflected the questions deemed most relevant to the implementation of the ACRE project. The scale reliabilities for the ten TDF scales were explored using Cronbach’s alpha, which provides the lower-bound estimate of the reliability of a test/measure. Relationships between the scales were explored using Pearson correlation coefficients.

**Table 1:** Implementation evaluation questions, TDF domains and descriptive statistics for the questionnaire items

Domain	Question		Means (SDs)
Knowledge	1	I know the objectives of the ACRE project	4.62 (0.52)
	2	The evidence that supports the ACRE pathway is strong	4.56 (0.62)
	3	I am aware of how the ACRE pathway is used in my hospital	4.69 (0.59)
Skills	4	The skills required to use the ACRE pathway are within the scope of an ED clinician	4.63 (0.58)
	5	The ACRE pathway is simple to use	4.45 (0.65)
	6	A junior ED doctor would have the capabilities to apply the ACRE pathway to a patient presenting with chest pain	4.16 (0.86)
Social/professional role and identity	7	Use of the ACRE pathway as a clinical decision rule is sound professional practice in the ED	4.35 (0.79)
	8	Having both ED and Cardiology specialists leading the ACRE project has helped improve acceptance of the project by local clinicians	4.45 (0.78)
	9	Clinicians from departments that manage patients with chest pain support the introduction of the ACRE project	4.21 (0.69)
Beliefs about capabilities	10	It is easy to utilise the ACRE pathway when the ED is busy	4.28 (0.67)
	11	The criteria of the ACRE pathway are clear to me	4.56 (0.56)
	12	I am confident I could apply the ACRE pathway to risk stratify a patient presenting to ED with chest pain	4.58 (0.53)
Optimism	13	I expect positive outcomes from the ACRE project	4.48 (0.67)
	14	I expect ACRE practices to be sustained beyond the completion of the ACRE project	4.46 (0.57)
	15	Overall, the ACRE project represents a positive change for Queensland Health	4.66 (0.54)
Beliefs about consequences	16	The ACRE project improves patient flow	4.57 (0.59)
	17	The ACRE project has improved management of patients presenting with chest pain	4.43 (0.65)
	18	The benefits from outcomes of the ACRE project will outweigh the time and effort required to adopt it	4.56 (0.59)
Intentions	19	I intend to use the ACRE pathway when appropriate to assess patients presenting with chest pain	4.65 (0.53)
	20	I intend to promote the education of future staff to utilise the ACRE pathway	4.71 (0.49)
Memory, attention and decision processes	21	Information in my workplace is useful to remind me to use the ACRE pathway	4.30 (0.76)
	22	Assessing a patient with chest pain triggers me to use the ACRE pathway	4.40 (0.79)
	23	If ACRE pathway letters and referrals are easily accessible I remember to use them	4.27 (0.66)
Environmental context and resources	24	The ACRE pathway is able to be adapted to local processes	4.42 (0.65)
	25	There has been sufficient local clinician time allocated to implement the ACRE pathway and processes	3.91 (0.96)
	26	There are good networks between parties involved in the adoption of the ACRE project	4.13 (0.85)
	27	Support from the ACRE project team has been integral to the successful implementation of the ACRE project	4.40 (0.68)
Social influences	28	Most people whose opinion I value would support the ACRE project	4.37 (0.71)
	29	My colleagues are supportive of the ACRE project	4.34 (0.73)
	30	Existing staff provide sufficient support to new staff to use the ACRE pathway	4.12 (0.76)

The questionnaire also asked the participants to indicate their employment site, and their professional group (i.e. medical, nursing or other, e.g. allied health). For ease of completion, and to encourage more respondents to complete the questionnaire, all questions were assessed using a Likert-scale response of 1 (strongly disagree) to 5 (strongly agree), with space for additional comments (see Additional file 1).

To test for differences in the scales on key variables (e.g. time since implementation, hospital size, staff groups), we used both repeated measures *t* tests with Bonferroni corrections and one-way ANOVAs.

### Participants

Questionnaires were distributed to clinician stakeholders who attended an ACRE project forum held in Brisbane in October 2015. Due to the staggered implementation across Queensland, timeframes from respondents implementing the ADAPT-ADP at their sites and completing the questionnaire vary markedly. Additionally, in November 2015, questionnaires were also mailed with a return envelope to other clinician stakeholders that had varying levels of involvement in the ACRE project. This included stakeholders that were identified by either their presence at initial introductory meetings and discussions about the project; were introduced to the project during the implementation phase due to their clinical role; had a role in implementing or educating on its introduction and use locally; or were senior and executive level clinicians who were regularly included in correspondence about the progress of the ACRE project at their site. This meant that we sent out a large number of questionnaires in the hope of getting a greater number and wider variety of responses, but potentially that the response *rate* would be lower as it was sent to people without a high level of direct engagement with the project. Two weeks after sending out the mailed questionnaires, stakeholders received a reminder email to complete the survey, which included a link and the option to complete an online version of the survey. A final reminder email was sent one more week after this. The chance to win one of three $50 gift cards was offered as incentive for those that returned the mailed or online questionnaire. Questionnaires were completed anonymously.

## Results

Sixty-three of 176 clinician stakeholders (36 %) responded to the questionnaire (25 from 31 forum attendees; 33 mailed and 5 online returns from 145) (see Additional file 2). Whilst the overall response rate was low, the number of responses is enough for our purposes. Nineteen hospital sites were represented by respondents, nine that had implemented the ACRE pathway for more than 12 months and ten that had implemented the ACRE pathway for less than 12 months and includes two sites that have not implemented at time of survey but have been introduced to project and planning stages. Respondent characteristics are listed in Table 2.

**Table 2:** Survey respondent characteristics

Time post-implementation	More than 12 months = 43 (68 %)
	Less than 12 months =20 (32 %)
Professional group	Medical = 29 (46 %)
	Nursing = 29 (46 %)
	Other/allied health = 5 (8 %)
Hospital size/type	Major metropolitan = 30 (48 %)
	Major regional = 23 (36 %)
	Large metropolitan = 7 (11 %)
	Medium sized = 3 (5 %)

*Major metropolitan* metropolitan hospitals with greater than 20,000 acute casemix-adjusted separations and greater than 20,000 emergency department presentations annually, *Major Regional* regional hospitals with greater than 16,000 acute casemix-adjusted separations and greater than 20,000 emergency department presentations annually, *Large metropolitan* metropolitan acute hospitals treating greater than 10,000 acute casemix-adjusted separations and greater than 20,000 emergency department presentations annually, *Medium sized* medium acute hospitals in metropolitan and regional areas treating between 5000 and 10,000 acute casemix-adjusted separations and greater than 20,000 emergency department resentations annually [19]

Table 1 also displays the means and standard deviations for all 30 items averaged across all participants. Responses to all questions were highly positive with small variance. Participants felt that they had the requisite knowledge and skills to implement the pathway in addition to it being within their professional identify and believing that the consequences would be positive. There are high levels of optimism for the ACRE project and thus intentions around implementation were very high. The three items with the lowest means (Q25, Q26, and Q30) highlight that perhaps one area for improvement could be the allocation of more resources and time locally to ensure the successful implementation.

All scales were shown to possess very good reliability (above 0.80 for seven scales and above 0.70 for three scales), and therefore, all items were combined into the appropriate ten scales for further analysis. Table 3 shows the means, standard deviations and reliability statistics for the ten scales.

**Table 3:** Descriptive statistics for the ten scales of the TDF

Scale (# Qs)	Mean (range)	SD	Scale reliability (*α*)
Knowledge (3)	4.61 (3–5)	0.49	0.76
Skills (3)	4.42 (2.67–5)	0.60	0.83
Social/professional role and identity (3)	4.34 (2.67–5)	0.60	0.71
Beliefs about capabilities (3)	4.47 (3–5)	0.53	0.87
Optimism (3)	4.53 (3–5)	0.53	0.87
Beliefs about consequences (3)	4.51 (3–5)	0.54	0.83
Intentions (2)	4.69 (3–5)	0.47	0.83
Memory, attention and decision processes (3)	4.30 (2.67–5)	0.65	0.80
Environmental context and resources (4)	4.23 (2.5–5)	0.62	0.81
Social influences (3)	4.23 (2–5)	0.68	0.75

The means for all scales show that, on average, the respondents are highly favourable to all aspects of the implementation. The *Social influences* scale shows the most variability and the lowest mean but still highly relative to the scale limits. The Intentions scale has the highest mean and lowest amount of variation in responses, indicating that intentions to use and promote the ACRE pathway was very high in the sample. The scale of *Social/professional role and identity* shows the lowest reliability with both the *Optimism* and *Beliefs about capabilities* scales possessing the highest reliabilities. All told, these data demonstrate that the instrument is reliable and that the items for each scale are measuring similar and related constructs. This provides support to the items and scales proposed by Huijg et al. [17]. Correlations between the ten scales were moderate to high, with most above 0.6. The *Skills* scale was least related to the others with lower bivariate correlations in the range of 0.26 to 0.5. The Intentions scale was the one most highly correlated to all of the others (range 0.66 to 0.75).

These ten scales were then used to look at differences in three key areas: (1) hospital staff, (2) time since implementation and (3) hospital size/type. Specifically, we tested if there were differences in responses to the 10 TDF scales between hospital sites that had implemented the ACRE pathway for more than 12 months versus those that had implemented the pathway for less than 12 months. There were no significant differences in 9 of the 10 scales for these two groupings of hospitals. However, there was a significant difference in the Social influences scale between hospitals and implementation time, *t*(59) = −2.56, *p* = 0.014, suggesting that staff reported receiving greater social support for the ACRE project in hospitals where the implementation had been in place for longer than 12 months.

We also tested for differences in professional group and found no significant differences between nursing and medical staff for any of the ten scales. Allied health workers were tested as a separate group in one set of analyses but because of the small sample size they were then grouped with nursing staff and analysis re-run. This made no difference to the conclusions. Hospitals were grouped according to size and location as per the Australian Institute of Health and Welfare hospital peer group codes (major metropolitan, major regional, large metropolitan, large regional, and medium) [19]. However, we found there were no significant differences in responses as a function of hospital type to any of the ten scales.

## Discussion

The translation of the ADAPT-ADP into clinical practice by the ACRE project was successful and is therefore a perfect case study for understanding factors which are important in driving implementation success. In some ways, the lack of variance in the responses hampered us from concluding which factors were really driving the success for the implementation. However, the overwhelmingly positive response also indicates that it was a successful programme and likely a combination of the factors that drove its success. Given the very high means, we can conclude that each of these domains was important for the implementation.

### Knowledge

The *Knowledge* scale had the second highest mean response and second least amount of variation in the responses. This indicates that knowledge and awareness of the ACRE project objectives, supporting evidence behind the pathway, and how the pathway is used in practice was strong. A key early step of introducing the ACRE project to each site was an education session describing the evidence base, as well as practical considerations of implementing the ACRE pathway at their hospital. Implementation efforts require consideration of factors beyond education strategies though, as Curran et al*.* [18] describe high levels of knowledge of the evidence and benefits behind the clinical decision rule they implemented, yet actual use in practice was low. However, ensuring high levels of knowledge still remains a vital step in implementing a new intervention and survey responses indicate that the ACRE project successfully did this.

### Skills

Responses in the Skills scale indicated that clinicians find the ACRE pathway simple to use, and they also believe their colleagues will find it simple to use. We believe that a key factor influencing successful implementation of the ACRE pathway is that it is simple to use in practice and is an adaptation, rather than replacement, of current pathways and guidelines commonly in use throughout Queensland. A comment highlighting the ease of use in response to Q6 was ‘All they need to do is use the available resources—they are readily available and clear to understand. If they read the step-by-step process, they can’t go wrong’.

### Social/professional role and identity

Although the Social/professional role and identity scale had a high mean response, some respondents’ comments provided insight into one of the potential barriers influencing successful implementation—the different levels of engagement and enthusiasm between different medical specialties to implement the project locally. Some examples include ‘Essential to have support of both teams’, ‘Minimal engagement from cardiology…’, ‘Need to improve links with cardiology team very much an ED project only…’, and ‘Little input locally from my in-patient colleagues…’. Whilst the ACRE project is very much an ‘ED pathway’, input and support from other relevant departments such as Cardiology, General Medicine, and Clinical Measurements was essential to ensure patient flow and adequate follow-up processes beyond the ED episode of care. Anecdotally, sites that had good engagement and relationships across departments reported easier implementation and earlier success than sites that did not. Early ACRE project meetings at sites aimed to include as many relevant departments and people as possible, to help create a shared vision and collaboration. However, it is possible that other departments did just not feel the same level of commitment to implementation of an ‘ED pathway’ or to prioritise it.

Some respondent comments also demonstrated some ambiguity around the interpretation of Q8, ‘Having both ED and Cardiology specialists leading the ACRE project has helped improve acceptance of the project by local clinicians’. The question related to the clinical leads of the ACRE project, whereas some respondents clearly answered this question in relation to their *local* ED and Cardiology specialists that were engaged as stakeholders at their sites. This might have affected the results or the scale reliability, but it is unclear how many responded in this way. This shows how comments can be useful to identify ambiguous questions which could be revised if the questionnaire was used again.

### Beliefs about capabilities

The Beliefs about capabilities scale showed that clinicians were positive about their ability to use the ACRE pathway in practice. Interestingly, Q10 (‘It is easy to utilise the ACRE pathway when the ED is busy’) had a slightly lower mean response than the other questions in this scale. A point was highlighted in Curran et al. [18] about conflicting views on whether use of their clinical decision rule actually improved or slowed patient flow through the ED when the department was busy. This might have also been relevant for the ACRE project, as one respondent stated, ‘Really depends on how ‘busy’ and what time of day…’. Despite the ACRE pathway being identified as simple to use and time saving, the extra thought processes to do something differently when it is busy and with multiple competing priorities may mean it is easier to revert back to previous standard practice. An important part of ensuring projects like this are implemented is making sure that the process is easy to follow and not complicated, or clinicians may revert back to standard practice that is already familiar. Furthermore, there was a slightly higher mean response to this question in sites that had implemented the ACRE pathway for >12 months versus <12 months, perhaps indicating that once the pathway is established and has become normal practice, it is easier to use when busy.

### Optimism

The Optimism scale had the third highest mean response and demonstrated that optimism around the ACRE project outcomes and sustainability in practice was high and therefore likely contributed as a positive factor influencing successful implementation. High levels of optimism around the project could relate back to a strong evidence base, positive outcomes achieved in the pilot study, and positive outcomes achieved in practice at their own sites, as well as optimism about sustaining it in practice. Clinicians may have also reported high levels of optimism as the ACRE pathway targets clinical redesign in a high-volume patient group and is therefore more likely to improve efficiency and produce ‘quick wins’ in high profile problem areas facing EDs such as patient flow and access and release capacity back into the system [20].

### Beliefs about consequences

Responses to questions in the scale *Beliefs about consequences* indicate that clinicians not only believe that the ACRE pathway improves patient flow and management of patients with possible cardiac chest pain, but also that the benefits from outcomes of the ACRE project will outweigh the time and effort required to adopt it (Q18). This is important, as there will be efforts and inevitable challenges involved when implementing change. All implementation efforts need to create this vision and belief that the work is worth doing, or implementation may fail, even when there is value in doing it [20].

### Intentions

The Intentions scale had the highest mean response and demonstrated the high intentions of respondents to both use the ACRE pathway and promote use and education to future staff. For example, ‘I will continue to promote pathway to our medical team’. This is positive feedback, as a person’s intentions are a strong predictor of behaviour [21]. However, predicting behaviours from intentions are also influenced by other factors such as social pressure [22] and therefore likely that ‘Intentions’ would be influenced by other domains in attempting to explain successful implementation and use of the pathway in practice.

### Memory, attention and decision processes

The *Memory, attention and decision processes* scale showed high responses; however, the mean response in this domain was lower than that in the Intentions domain. This suggests that despite high intentions to use a new pathway or decision-making tool in practice, it needs to be easy to use and easily accessible to promote use. Steps taken to achieve this included local project officers or engaged stakeholders at sites providing education, placing posters up, and ensuring that the pathways and associated documents were available and easily accessible. To promote use of the pathway at one site, it was placed with chest pain patients as soon as they arrived in ED by the triage nurse. This idea was shared with other sites, some of which also adopted this approach and reported improved use of the pathway. It remains a challenge though, as several comments highlight, ‘One of the acknowledged challenges in a department full of forms and paperwork is having the paperwork stand out’ and ‘Need to maintain availability as new staff members join team’. This may improve once the new intervention has been in place for a period of time and is embedded into usual practice though, as mean responses for questions in this domain were slightly higher in sites that had implemented for >12 months. As one respondent commented, ‘Is already well ingrained in practice’*.*

### Environmental context and resources

The Environmental context and resources scale had the equal lowest mean response. It included questions about external factors we thought were likely to influence implementation. Q24 examined one of the features that we felt was a key factor influencing successful implementation, especially across many different sites, flexibility of the pathway and adapting it to fit with local processes and preferences, rather than a standard approach to implementation across all sites. This may also help explain why there was no significant difference in responses between stakeholders by hospital size and type. The hospitals that have implemented the pathway vary widely, from major metropolitan hospitals with cardiology departments to medium-sized and regional hospitals without any internal cardiology services for following up patients. Adaptability of an intervention to suit the local environment has been shown to be an important factor influencing implementation [23, 24]. Tailoring the ACRE pathway to fit local practice, for example, flexibility with outpatient and follow up processes, helped enable successful implementation across multiple different sites.

Questions 25 and 26 explored if respondents felt that there had been sufficient local clinician time allocated to implement the ACRE pathway and processes and if there are good networks between parties involved in the adoption of the ACRE project. Expectedly, these questions had lower mean responses than the majority of questions, and Q25 was the item with the lowest mean score overall. Whilst most organisations would agree that evidence-based innovative changes to practice that produce positive outcomes are beneficial, it may be more difficult to achieve in practice without the available resources, especially around staffing availability. The ACRE project offered a limited amount of funding to sites to support a local clinician (e.g. for the provision of offline time, or extra shifts) to work across local aspects of implementation. However, this was rarely accepted as sites found it difficult to spare someone the time, and local work was often done by a clinician around their normal work role. Sites that were able to make use of this funding for dedicated time towards local implementation of the project benefitted greatly. Q26 explored local networks and highlights that better collaboration and involvement across departments help implementation efforts. Similar points have been discussed under Social/professional role and identity. To highlight these points further, Q26 comments include, ‘Would not have started without team liaising with and convincing local cardiologist to use it’ and ‘ACRE mostly adapted unilaterally by ED with some cardiology agreement’.

### Social influences

The Social influences scale shows the lowest mean (with Environmental context and resources) and the most variability, but responses overall were still very positive. Interestingly, this was the only scale to show a significant difference between hospitals by implementation time, suggesting that wider support for the ACRE project improved over time as the pathway became embedded into routine practice. This aligns with Rogers ‘diffusion of innovations’ model in which there is some lag time before the ‘early majority’ and then the ‘late majority’ supports or adopt a new innovation [25]. This scale also links to a factor that we believe contributed to success—the order in which sites were approached to implement the project. There were several sites we felt would be more receptive to implementation and more likely to be engaged with the project team than others, for example, sites that approached us to express interest in the project or, simply based on our own perceptions of sites that may more readily implement change in this field, from knowledge working within the organisation. These sites were targeted first to try and achieve early success. This helped garner momentum for the project and drive implementation at subsequent sites, who may have also felt pressure to implement once other peer or competing hospitals had already implemented [26].

The item with the second lowest mean score overall was Q30, ‘Existing staff provide sufficient support to new staff to use the ACRE pathway’. Two comments further highlighted this challenge, ‘Orientation of new ED staff is very challenging given: large number of processes across multiple areas need to be included, this is just one; no single orientation time for all new staff given shift work; orientation once appears insufficient; staff need to be supported on floor when initially using the pathway in order to avoid basic errors…’ and ‘Our department has multiple part-time senior clinicians and a very transient SHO/RMO group—challenges in keeping the message consistent and current’. This should be considered, especially in regard to planning for sustainability, as implementation is less likely to be successful in organisations with high staff turnover [27].

### Scale reliability

These data demonstrate that the TDF is a useful tool for designing specific questions to evaluate implementation behaviours. Our instrument was shown to be reliable in so far as the items for each scale are measuring similar and related constructs. This provides support to the items and scales proposed by Huijg et al*.* [17], which formed the basis for our questionnaire. All scales demonstrated good reliability, and are promising for use in future research, such as further investigating the concurrent and predictive validity and reliability of the questionnaire in practice [17].

We tailored the instrument to suit our purposes and purposefully excluded one domain (Emotion) from the overall questionnaire based on relevance. The rationale for leaving out scales is specific to the nature of the programme and therefore needs to be judged on a case-by-case basis. The TDF is a useful framework for evaluation but is very broad and encompassing and therefore it is likely to require tailoring or shaping to the local implementation context. We have demonstrated how this can be successfully achieved.

### Limitations

This study had a few limitations. The questionnaire was not pre-tested to establish feasibility before using it for the principal data collection phase. This was primarily for logistical reasons including time constraints and a lack of a relevant sample group to enable such testing to take place. With a larger implementation project, a small scale pre-test is advisable. We acknowledge that the questionnaire results may have been affected by several factors, influencing respondent bias. First, it is likely that the overwhelmingly positive responses to the survey questions were at least partly due to the stakeholders most engaged with and positive about the ACRE project being the ones who completed the survey. Furthermore, social desirability bias may have influenced results. Each clinician that completed the survey has had prior communication and interaction with the ACRE project team and therefore may have been more likely to give us positive feedback. However, it is our experience that medical specialists and clinicians are not averse to reporting their true feelings on the nature of an intervention that directly affects them. Moreover, the surveys were anonymous and any reputation effects or concern for negative impact would be largely mitigated by this. Additionally, accuracy of some responses may have been influenced by recall bias, as some respondents were completing the survey more than 12 months after the initial implementation period, despite regular and ongoing (e.g. at least once monthly) email correspondence from the project team. The option of a ‘Don’t know/NA’ response for each survey question was designed to limit this bias.

High mean responses across all domains made it difficult to gauge their relative importance to implementation outcomes and limited the ability to explore if there are any important interactions between the domains. Interviews, follow-up questions or a different survey design may have helped answer these questions; however, questionnaires that were simple to complete were used as they enabled us to sample a larger number of stakeholders. Additionally, despite broadly encompassing factors using the TDF, the questions used were limited by our selection of determinants and therefore may not have captured all relevant factors influencing implementation. Other factors, beyond the scope of the TDF questions, would also have impacted on the success of the project. For example, macro organisational factors such as readiness to adopt (organisational culture) and political pressures on hospitals to reduce waiting times in EDs are systemic factors linked to implementation success. However, it is difficult to assess their real impact at the local level, and we therefore focused our efforts on examining local stakeholders’ responses in regard to their own local implementation.

Finally, whilst we report the ACRE project as being widely and successfully implemented across 19 sites, there were different levels of ‘success’ and engagement between sites, for example, the time and effort taken to implement into practice, and use and uptake of the ACRE pathway in practice varied. Further examination between sites that varied in ‘success’ by these measures did not reveal any major variation in responses. However, rather than a fault of the TDF or questionnaire to differentiate this, we were limited by low (or nil) responses from several sites that we felt were not as ‘successful’ in implementation. Similarly, low numbers of responses in several categories for statistical comparisons, such as hospital type and size, limit generalizability of the findings.

## Conclusions

The TDF was used to guide questionnaire development to evaluate the factors driving the highly successful implementation of the ACRE project in multiple sites across Queensland with varying characteristics. Stakeholder survey results were overwhelmingly positive, with high mean responses across all scales, demonstrating that the implementation strategy was effective. However, whilst the domains did not appear very discriminatory and a lack of variance in the responses hampered us from concluding which factors were really driving success, they did match positive outcomes with positive responses and so could therefore be used as a starting point for assessing future projects of a similar nature.

The TDF offers a useful framework to conceptualise and evaluate factors impacting on implementation success. However, its broad scope makes it necessary to tailor and adapt it to the specific purpose of question at hand. This had advantages and disadvantages. It places a large burden on researchers, often lacking in psychometric expertise, to develop their own questions. Future research could focus on the rigorous systematic development of scales or instruments to assess implementation success both pre- and post-implementation. This may help to determine which factors actually influence success and would benefit from action, as well as help specify any relationships between the domains. It would also enable the comparison of findings across studies, which is difficult to achieve without standardised scales.

## Additional files

Additional file 1: ACRE project implementation evaluation questionnaire. (DOC 105 kb)
Additional file 2: Collated survey responses. (XLSX 22 kb)

## Abbreviations

ACRE: Accelerated Chest pain Risk Evaluation
ADAPT: 2-Hour Accelerated Diagnostic Protocol to Assess Patients With Chest Pain Symptoms Using Contemporary Troponins as the Only Biomarker
ADP: Accelerated diagnostic protocol
EBP: Evidence-based practice
ED: Emergency departments
TDF: Theoretical Domains Framework
