# Supplementary material for: Use of the Theoretical Domains Framework to evaluate factors driving successful implementation of the Accelerated Chest pain Risk Evaluation (ACRE) project
Source: Implement Sci. 2016 Oct 12;11:136. doi: 10.1186/s13012-016-0500-9 (PMC5062925; doi:10.1186/s13012-016-0500-9)
Supplement: Additional file 1: — ACRE project implementation evaluation questionnaire. (DOC 105 kb) [file 13012_2016_500_MOESM1_ESM.doc]

**Accelerated Chest pain Risk Evaluation (ACRE) Project: Implementation evaluation - stakeholder survey**

If you agree to take part, please complete and return the questionnaire. The return of the completed questionnaire will be taken as agreement for your information to be used in this project.

Reporting or publishing of results and information you submit will not be identifiable in any way.

Workplace (*hospital name only):* ________________________________________________________

Professional Group (*e.g. medical, nursing, allied health*): _____________________________________

Please note:

- ‘*ACRE Pathway’* refers to the actual chest pain pathway adopted by your local site including the Accelerated Diagnostic Protocol (ADP) criteria, and associated follow-on processes
- ‘*ACRE Project’* refers to the overall, temporary entity (including the ACRE project team) that has been created to introduce the ACRE Pathway into practice

| 1. I know the objectives of the ACRE Project | | | | | |
| --- | --- | --- | --- | --- | --- |
| **Strongly Disagree**  **1** | **Disagree**  **2** | **Neutral**  **3** | **Agree**  **4** | **Strongly Agree**  **5** | **Don’t know / NA**  **NA** |
| ________________________________________________________________________________________________________________________________________________________________________________________________________________________________________________________________________________________________________________________________________________________________ | | | | | |
| 1. The evidence that supports the ACRE Pathway is strong | | | | | |
| **Strongly Disagree**  **1** | **Disagree**  **2** | **Neutral**  **3** | **Agree**  **4** | **Strongly Agree**  **5** | **Don’t know / NA**  **NA** |
| ________________________________________________________________________________________________________________________________________________________________________________________________________________________________________________________________________________________________________________________________________________________________ | | | | | |
| 1. I am aware of how the ACRE Pathway is used in my hospital | | | | | |
| **Strongly Disagree**  **1** | **Disagree**  **2** | **Neutral**  **3** | **Agree**  **4** | **Strongly Agree**  **5** | **Don’t know / NA**  **NA** |
| ________________________________________________________________________________________________________________________________________________________________________________________________________________________________________________________________________________________________________________________________________________________________ | | | | | |
| 1. The skills required to use the ACRE Pathway are within the scope of an ED clinician | | | | | |
| **Strongly Disagree**  **1** | **Disagree**  **2** | **Neutral**  **3** | **Agree**  **4** | **Strongly Agree**  **5** | **Don’t know / NA**  **NA** |
| ________________________________________________________________________________________________________________________________________________________________________________________________________________________________________________________________________________________________________________________________________________________________ | | | | | |
| 1. The ACRE Pathway is simple to use | | | | | |
| **Strongly Disagree**  **1** | **Disagree**  **2** | **Neutral**  **3** | **Agree**  **4** | **Strongly Agree**  **5** | **Don’t know / NA**  **NA** |
| ________________________________________________________________________________________________________________________________________________________________________________________________________________________________________________________________________________________________________________________________________________________________ | | | | | |
| 1. A junior ED doctor would have the capabilities to apply the ACRE Pathway to a patient presenting with chest pain | | | | | |
| **Strongly Disagree**  **1** | **Disagree**  **2** | **Neutral**  **3** | **Agree**  **4** | **Strongly Agree**  **5** | **Don’t know / NA**  **NA** |
| ________________________________________________________________________________________________________________________________________________________________________________________________________________________________________________________________________________________________________________________________________________________________ | | | | | |
| 1. Use of the ACRE Pathway as a clinical decision rule is sound professional practice in the ED | | | | | |
| **Strongly Disagree**  **1** | **Disagree**  **2** | **Neutral**  **3** | **Agree**  **4** | **Strongly Agree**  **5** | **Don’t know / NA**  **NA** |
| ________________________________________________________________________________________________________________________________________________________________________________________________________________________________________________________________________________________________________________________________________________________________ | | | | | |
| 1. Having both ED and Cardiology specialists leading the ACRE Project has helped improve acceptance of the Project by local clinicians | | | | | |
| **Strongly Disagree**  **1** | **Disagree**  **2** | **Neutral**  **3** | **Agree**  **4** | **Strongly Agree**  **5** | **Don’t know / NA**  **NA** |
| ________________________________________________________________________________________________________________________________________________________________________________________________________________________________________________________________________________________________________________________________________________________________ | | | | | |
| 1. Clinicians from departments that manage patients with chest pain support the introduction of the ACRE Project | | | | | |
| **Strongly Disagree**  **1** | **Disagree**  **2** | **Neutral**  **3** | **Agree**  **4** | **Strongly Agree**  **5** | **Don’t know / NA**  **NA** |
| ________________________________________________________________________________________________________________________________________________________________________________________________________________________________________________________________________________________________________________________________________________________________ | | | | | |
| 1. It is easy to utilise the ACRE Pathway when the ED is busy | | | | | |
| **Strongly Disagree**  **1** | **Disagree**  **2** | **Neutral**  **3** | **Agree**  **4** | **Strongly Agree**  **5** | **Don’t know / NA**  **NA** |
| ________________________________________________________________________________________________________________________________________________________________________________________________________________________________________________________________________________________________________________________________________________________________ | | | | | |
| 1. The criteria of the ACRE Pathway are clear to me | | | | | |
| **Strongly Disagree**  **1** | **Disagree**  **2** | **Neutral**  **3** | **Agree**  **4** | **Strongly Agree**  **5** | **Don’t know / NA**  **NA** |
| ________________________________________________________________________________________________________________________________________________________________________________________________________________________________________________________________________________________________________________________________________________________________ | | | | | |
| 1. I am confident I could apply the ACRE Pathway to risk stratify a patient presenting to ED with chest pain | | | | | |
| **Strongly Disagree**  **1** | **Disagree**  **2** | **Neutral**  **3** | **Agree**  **4** | **Strongly Agree**  **5** | **Don’t know / NA**  **NA** |
| ________________________________________________________________________________________________________________________________________________________________________________________________________________________________________________________________________________________________________________________________________________________________ | | | | | |
| 1. I expect positive outcomes from the ACRE Project | | | | | |
| **Strongly Disagree**  **1** | **Disagree**  **2** | **Neutral**  **3** | **Agree**  **4** | **Strongly Agree**  **5** | **Don’t know / NA**  **NA** |
| ________________________________________________________________________________________________________________________________________________________________________________________________________________________________________________________________________________________________________________________________________________________________ | | | | | |
| 1. I expect ACRE practices to be sustained beyond the completion of the ACRE Project | | | | | |
| **Strongly Disagree**  **1** | **Disagree**  **2** | **Neutral**  **3** | **Agree**  **4** | **Strongly Agree**  **5** | **Don’t know / NA**  **NA** |
| ________________________________________________________________________________________________________________________________________________________________________________________________________________________________________________________________________________________________________________________________________________________________ | | | | | |
| 1. Overall, the ACRE Project represents a positive change for Queensland Health | | | | | |
| **Strongly Disagree**  **1** | **Disagree**  **2** | **Neutral**  **3** | **Agree**  **4** | **Strongly Agree**  **5** | **Don’t know / NA**  **NA** |
| ________________________________________________________________________________________________________________________________________________________________________________________________________________________________________________________________________________________________________________________________________________________________ | | | | | |
| 1. The ACRE Project improves patient flow | | | | | |
| **Strongly Disagree**  **1** | **Disagree**  **2** | **Neutral**  **3** | **Agree**  **4** | **Strongly Agree**  **5** | **Don’t know / NA**  **NA** |
| ________________________________________________________________________________________________________________________________________________________________________________________________________________________________________________________________________________________________________________________________________________________________ | | | | | |
| 1. The ACRE Project has improved management of patients presenting with chest pain | | | | | |
| **Strongly Disagree**  **1** | **Disagree**  **2** | **Neutral**  **3** | **Agree**  **4** | **Strongly Agree**  **5** | **Don’t know / NA**  **NA** |
| ________________________________________________________________________________________________________________________________________________________________________________________________________________________________________________________________________________________________________________________________________________________________ | | | | | |
| 1. The benefits from outcomes of the ACRE Project will outweigh the time and effort required to adopt it | | | | | |
| **Strongly Disagree**  **1** | **Disagree**  **2** | **Neutral**  **3** | **Agree**  **4** | **Strongly Agree**  **5** | **Don’t know / NA**  **NA** |
| ________________________________________________________________________________________________________________________________________________________________________________________________________________________________________________________________________________________________________________________________________________________________ | | | | | |
| 1. I intend to use the ACRE Pathway when appropriate to assess patients presenting with chest pain | | | | | |
| **Strongly Disagree**  **1** | **Disagree**  **2** | **Neutral**  **3** | **Agree**  **4** | **Strongly Agree**  **5** | **Don’t know / NA**  **NA** |
| ________________________________________________________________________________________________________________________________________________________________________________________________________________________________________________________________________________________________________________________________________________________________ | | | | | |
| 1. I intend to promote the education of future staff to utilise the ACRE Pathway | | | | | |
| **Strongly Disagree**  **1** | **Disagree**  **2** | **Neutral**  **3** | **Agree**  **4** | **Strongly Agree**  **5** | **Don’t know / NA**  **NA** |
| ________________________________________________________________________________________________________________________________________________________________________________________________________________________________________________________________________________________________________________________________________________________________ | | | | | |
| 1. Information in my workplace is useful to remind me to use the ACRE Pathway | | | | | |
| **Strongly Disagree**  **1** | **Disagree**  **2** | **Neutral**  **3** | **Agree**  **4** | **Strongly Agree**  **5** | **Don’t know / NA**  **NA** |
| ________________________________________________________________________________________________________________________________________________________________________________________________________________________________________________________________________________________________________________________________________________________________ | | | | | |
| 1. Assessing a patient with chest pain triggers me to use the ACRE Pathway | | | | | |
| **Strongly Disagree**  **1** | **Disagree**  **2** | **Neutral**  **3** | **Agree**  **4** | **Strongly Agree**  **5** | **Don’t know / NA**  **NA** |
| ________________________________________________________________________________________________________________________________________________________________________________________________________________________________________________________________________________________________________________________________________________________________ | | | | | |
| 1. If ACRE Pathway letters and referrals are easily accessible I remember to use them | | | | | |
| **Strongly Disagree**  **1** | **Disagree**  **2** | **Neutral**  **3** | **Agree**  **4** | **Strongly Agree**  **5** | **Don’t know / NA**  **NA** |
| ________________________________________________________________________________________________________________________________________________________________________________________________________________________________________________________________________________________________________________________________________________________________ | | | | | |
| 1. The ACRE Pathway is able to be adapted to local processes | | | | | |
| **Strongly Disagree**  **1** | **Disagree**  **2** | **Neutral**  **3** | **Agree**  **4** | **Strongly Agree**  **5** | **Don’t know / NA**  **NA** |
| ________________________________________________________________________________________________________________________________________________________________________________________________________________________________________________________________________________________________________________________________________________________________ | | | | | |
| 1. There has been sufficient local clinician time allocated to implement the ACRE Pathway and processes | | | | | |
| **Strongly Disagree**  **1** | **Disagree**  **2** | **Neutral**  **3** | **Agree**  **4** | **Strongly Agree**  **5** | **Don’t know / NA**  **NA** |
| ________________________________________________________________________________________________________________________________________________________________________________________________________________________________________________________________________________________________________________________________________________________________ | | | | | |
| 1. There are good networks between parties involved in the adoption of the ACRE Project | | | | | |
| **Strongly Disagree**  **1** | **Disagree**  **2** | **Neutral**  **3** | **Agree**  **4** | **Strongly Agree**  **5** | **Don’t know / NA**  **NA** |
| ________________________________________________________________________________________________________________________________________________________________________________________________________________________________________________________________________________________________________________________________________________________________ | | | | | |
| 1. Support from the ACRE Project team has been integral to the successful implementation of the ACRE Project | | | | | |
| **Strongly Disagree**  **1** | **Disagree**  **2** | **Neutral**  **3** | **Agree**  **4** | **Strongly Agree**  **5** | **Don’t know / NA**  **NA** |
| ________________________________________________________________________________________________________________________________________________________________________________________________________________________________________________________________________________________________________________________________________________________________ | | | | | |
| 1. Most people whose opinion I value would support the ACRE Project | | | | | |
| **Strongly Disagree**  **1** | **Disagree**  **2** | **Neutral**  **3** | **Agree**  **4** | **Strongly Agree**  **5** | **Don’t know / NA**  **NA** |
| ________________________________________________________________________________________________________________________________________________________________________________________________________________________________________________________________________________________________________________________________________________________________ | | | | | |
| 1. My colleagues are supportive of the ACRE Project | | | | | |
| **Strongly Disagree**  **1** | **Disagree**  **2** | **Neutral**  **3** | **Agree**  **4** | **Strongly Agree**  **5** | **Don’t know / NA**  **NA** |
| ________________________________________________________________________________________________________________________________________________________________________________________________________________________________________________________________________________________________________________________________________________________________ | | | | | |
| 1. Existing staff provide sufficient support to new staff to use the ACRE Pathway | | | | | |
| **Strongly Disagree**  **1** | **Disagree**  **2** | **Neutral**  **3** | **Agree**  **4** | **Strongly Agree**  **5** | **Don’t know / NA**  **NA** |
| ________________________________________________________________________________________________________________________________________________________________________________________________________________________________________________________________________________________________________________________________________________________________ | | | | | |

***The ACRE Team wishes to thank you for your time and support***
